# Editorial: Maternal PTSD, complex trauma, post-traumatic growth and resiliency across different cultures

**DOI:** 10.3389/fgwh.2026.1822585

**Published:** 2026-04-01

**Authors:** Maria Muzik, Geetha Shivakumar, Catherine Chamberlain, Prabha S. Chandra

**Affiliations:** 1 Departments of Psychiatry and Obstetrics & Gynecology Faculty, Center for Global Health Equity Faculty, Global REACH University of Michigan Medical School Michigan Medicine, Ann Arbor, MI, United States; 2 Department of Psychiatry, UT Southwestern Medical Center, and Staff Psychiatrist, Dallas VA Medical Center, Dallas, TX, United States; 3 Melbourne School of Population and Global Health Melbourne, University of Melbourne, Melbourne, VIC, Australia; 4 Department of Psychiatry National Institute of Mental Health and Neurosciences, Bangalore, India

**Keywords:** attachment, interventions, maternal trauma, parenting, PTSD

Maternal trauma, in all its forms, casts a long and intricate shadow over generations, cultures, and health outcomes. The significance of this issue becomes particularly clear in the context of pregnancy and early parenting, where the prevalence and impact of post-traumatic stress disorder (PTSD) and complex trauma are far from trivial. In the general population, rates are estimated at 3%–4%, but among women experiencing high-risk pregnancies or have been impacted by trauma and oppression, these numbers jump dramatically to 18%–20%. This special issue of *Frontiers in Global Women's Health* goes beyond the numbers, presenting research that brings to life the lived experiences, intergenerational effects, and pathways toward resilience and healing, as well as the potential for innovative treatment approaches.

At the heart of this Research Topic lies an understanding that trauma's negative effects are profoundly multifaceted. Trauma not only affects individual mothers but also ripples outward, touching their children and entire communities. The mechanisms by which maternal trauma is transmitted intergenerationally—often following universal pathways—are fundamental targets for intervention and can be studied and addressed through a global lens.

Eunjeong Cho and colleagues offer a compelling exploration of how adverse childhood experiences (ACEs) among mothers affect their own children's quality of life. Their paper, drawing on a national survey from South Korea, demonstrates how the mother's own psychological health and self-esteem mediate these effects. Importantly, they find that maternal self-esteem moderates the risk transmission, highlighting a powerful point of intervention: programs that enhance maternal psychological wellbeing and empowerment could help break cycles of adversity and foster positive outcomes for both mothers and children. This insight underscores an urgent need for trauma-informed, family-centered approaches that address both symptoms and structural causes of trauma.

Building on the intergenerational model, Sarah Freedman and colleagues examine data collected in the United States to shed light on the mediating role of parent emotion regulation in the link between mothers' ACEs and their children's emotion regulation. Their work suggests that intervention strategies targeting parents’ own capacities for emotion regulation—not just generic parenting or emotion-related socialization skills—may be crucial for breaking cycles of trauma and fostering healthy emotional development in the next generation. The takeaway is clear: supporting parental regulation is foundational for positive child outcomes.

Sarah M. Ahlfs-Dunn and her team turn the discussion toward the importance of early identification of risk. Their study highlights how prenatal PTSD symptoms in mothers can disrupt maternal representations of their child, leading to difficulties in infant social-emotional functioning. The findings point to the necessity of routine prenatal PTSD screening and the value of early intervention, even before birth, when disrupted maternal representations are apparent. By focusing on the prenatal period, this research offers a critical window for prevention, making the case for holistic, trauma-informed care that is attentive to both mother and child.

Geneviève Lapointe and colleagues, using data from Canada, further underscore the impact of complex trauma on pregnant women's mental health and functioning. Specifically, they show that developmental trauma disorder (DTD) among expectant mothers is linked to more severe PTSD symptoms, diminished maternal competence, disrupted prenatal attachment, and greater risk for co-occurring mental health disorders. Their findings highlight the distinctions between women who experienced maltreatment but did not develop DTD and those who did, reinforcing the essential need for early intervention, nuanced assessment, and tailored treatment approaches.

Of course, even the most promising clinical or social support interventions can be undermined by very real barriers. Many women face internal obstacles—like shame, helplessness, or distrust of providers—as well as external impediments, such as lack of transportation, childcare, or previous negative experiences in healthcare settings. Effective trauma-informed programs must prioritize emotional safety, flexibility, and respect, creating what might be called a “soft-safe entry” into care. This principle is emphasized repeatedly by both research and clinical practice.

Phyllis Raynor and colleagues turn our attention to maternal grief and shame in the context of addiction recovery, using a community-based participatory approach among women in the United States. Their research reveals that loss events—from the loss of custody to loss of attachment—are profoundly associated with grief and shame among pregnant and early parenting women. The intense stigma surrounding maternal substance use disorders calls for supportive, non-judgmental environments that directly address these complicated emotions as central to the recovery process.

Similarly, Anisha Devi Bhagawan and her team provide a qualitative exploration of birth trauma among women in Ireland. Their study illuminates the immense psychological burden and stigma associated with traumatic birth experiences. Key themes—such as feeling personally failed, or failed by others, and experiencing threats to life—underscore the need for trauma-informed, woman-centered practices within health services. By centering women's own voices, this work advocates for a shift toward empathy, reflection, and sensitive responsiveness in maternity care.

Joanna Koutanis and colleagues investigate an innovative pharmacological approach to preventing postpartum child-birth PTSD: the use of glucocorticoids immediately after birth trauma. While their pilot trial—using a US sample—did not observe a reduction in PTSD symptomatology, the study established the feasibility and acceptability of enrolling patients in the immediate postpartum period. This sets the stage for future, larger trials and demonstrates the importance of multidimensional approaches to treatment.

Bringing together the findings across studies, this Research Topic emphasizes the urgent need for trauma-informed care at all levels of maternal health. Approaches like the Common Elements Treatment Approach (CETA; Murray et al.) or Mom Power (MP; Rosenblum et al.) offer robust, transdiagnostic and multi-generational frameworks that address trauma, depression, anxiety, PTSD, and substance use. CETA integrates cognitive-behavioral techniques appropriate for low-resource and high-conflict settings (Murray et al.; Murray et al.), while Mom Power combines attachment theory and evidence-based infant mental health practices to enhance parental resilience, parenting capacity, reflective functioning, and empathic attunement (Rosenblum et al.; Forer et al.). Critically, both programs address systemic and individual barriers to care, fostering environments that scaffold emotional safety, a non-judgmental stance, and empowerment for parents. Ultimately, what is needed worldwide are universally accessible, trauma-informed solutions that prioritize hope, growth, and healing for entire families. By shifting the narrative from risk transmission and deficit to one of empowerment and recovery, we can break cycles of trauma and transform children's wellbeing across generations and cultures.

